# Sodium MRI of the Lumbar Intervertebral Discs of the Human Spine: An Ex Vivo Study

**DOI:** 10.1002/jmri.29521

**Published:** 2024-07-04

**Authors:** Benedikt Kamp, Karl Ludger Radke, Marek Knet, Rosanna Strunk, Patrik J. Gallinnis, Armin M. Nagel, Timm J. Filler, Gerald Antoch, Daniel B. Abrar, Miriam Frenken, Hans‐Jörg Wittsack, Anja Müller‐Lutz

**Affiliations:** ^1^ Department of Diagnostic and Interventional Radiology Medical Faculty and University Hospital Düsseldorf, Heinrich‐Heine‐University Düsseldorf Düsseldorf Germany; ^2^ Institute of Radiology, University Hospital Erlangen Friedrich‐Alexander‐Universität Erlangen‐Nürnberg (FAU) Erlangen Germany; ^3^ Division of Medical Physics in Radiology German Cancer Research Center (DKFZ) Heidelberg Germany; ^4^ Institute of Anatomy I Heinrich‐Heine‐University Düsseldorf Düsseldorf Germany

**Keywords:** sodium MRI, ^23^Na MRI, ^23^Na relaxation times, intervertebral disc, IVD, ex vivo

## Abstract

**Background:**

Lower back pain affects 75%–85% of people at some point in their lives. The detection of biochemical changes with sodium (^23^Na) MRI has potential to enable an earlier and more accurate diagnosis.

**Purpose:**

To measure ^23^Na relaxation times and apparent tissue sodium concentration (aTSC) in ex‐vivo intervertebral discs (IVDs), and to investigate the relationship between aTSC and histological Thompson grade.

**Study Type:**

Ex‐vivo.

**Specimen:**

Thirty IVDs from the lumbar spines of 11 human body donors (4 female, 7 male, mean age 86 ± 8 years).

**Field Strength/Sequence:**

3 T; density‐adapted 3D radial sequence (DA‐3D‐RAD).

**Assessment:**

IVD ^23^Na longitudinal (*T*_1_), short and long transverse (*T*_2s_* and *T*_2l_*) relaxation times and the proportion of the short transverse relaxation (*p*_*s*_) were calculated for one IVD per spine sample (11 IVDs). Furthermore, aTSCs were calculated for all IVDs. The degradation of the IVDs was assessed via histological Thompson grading.

**Statistical Tests:**

A Kendall Tau correlation (*τ*) test was performed between the aTSCs and the Thompson grades. The significance level was set to *P* < 0.05.

**Results:**

Mean ^23^Na relaxation parameters of a subset of 11 IVDs were *T*_1_ = 9.8 ± 1.3 msec, *T*_2s_* = 0.7 ± 0.1 msec, *T*_2l_* = 7.3 ± 1.1 msec, and *p*_*s*_ = 32.7 ± 4.0%. A total of 30 IVDs were examined, of which 3 had Thompson grade 1, 4 had grade 2, 5 had grade 3, 5 had grade 4, and 13 had grade 5. The aTSC decreased with increasing degradation, being 274.6 ± 18.9 mM for Thompson grade 1 and 190.5 ± 29.5 mM for Thompson grade 5. The correlation between whole IVD aTSC and Thompson grade was significant and strongly negative (*τ* = −0.56).

**Data Conclusion:**

This study showed a significant correlation between aTSC and degenerative IVD changes. Consequently, aTSC has potential to be useful as an indicator of degenerative spinal changes.

**Evidence Level:**

2

**Technical Efficacy:**

Stage 1

Lower back pain (LBP) affects 75% to 85% of people at some point in their lives.^1^ The leading cause for LBP is degenerative disc disease (DDD) of the intervertebral disc (IVD).^2^ The IVD can be subdivided into an inner gelatinous and proteoglycan (PG) rich nucleus pulposus (NP) and an outer multi‐lamellar and collagen rich annulus fibrosus (AF).^3^ PGs play a major role in the ability of the NP to hold water, and during DDD the PG content decreases.^4^ The resulting water loss in the NP leads to a gradual decrease of resilience against mechanical stress.^4^ In addition, because the collagen matrix of the AF deteriorates during DDD, the consequence can be bulging and a general loss of disc height.^4^

While the imaging standard for diagnosing DDD is MRI, most classification is based on morphological assessment of the IVD, detecting factors like signal loss in the NP on *T*_2_ weighted images, disc herniation or a general decrease in height of the IVD.^2^, ^5^ However, biochemical changes of the IVD, including in the collagen matrix, precede morphological changes.^5^, ^6^ Therefore, the detection of biochemical changes may enable an earlier and more accurate diagnosis.^7^

A number of compositional quantitative techniques have been proposed to detect the biochemical changes using MRI. *T*_2_* mapping can be used to detect changes in the collagen matrix, and *T*_2_ mapping can detect water content in the IVD.^8^ Furthermore, there are techniques that have been shown to be more specific to changes in PG content, such as glycosaminoglycan (GAG) chemical exchange saturation transfer (CEST), *T*_1ρ_ mapping and sodium (^23^Na) imaging.^9^, ^10^, ^11^ Previous studies have examined the feasibility of ^23^Na MRI for the lumbar spine in a rabbit model and with multi‐array coils in vivo.^12^, ^13^, ^14^

^23^Na imaging is sensitive to PG content because ^23^Na ions accumulate on the negatively charged GAG side chains of PG.^15^ Thus, a loss of tissue sodium concentration (TSC) in the IVD reflects a loss of PG, which occurs in the early stages of degeneration of the IVD. However, ^23^Na imaging can be challenging due to a number of factors. In the human body, ^23^Na is approximately 2000 times rarer than hydrogen (^1^H), and is only approximately 9% as sensitive to magnetic resonance.^16^ Furthermore, ^23^Na exhibits a biexponential transversal relaxation process with a very short relaxation time component, necessitating the use of ultra‐short echo time imaging sequences.^17^

These factors can lead to lengthy ^23^Na imaging protocols which are difficult to apply in vivo. Even at the lower imaging resolution of ^23^Na MRI, longer protocols are necessary to achieve an acceptable signal‐to‐noise ratio (SNR).^16^ Also, for a precise determination of the TSCs, the measurement of the ^23^Na relaxation times is necessary.^16^ Relaxation time measurements require multiple acquisitions with varying imaging parameters, further extending the protocol duration. Such long imaging protocols are less problematic for ex vivo samples. It should also be noted that, when assessing TSC with MRI, as the signal loss resultant from other factors like pulse sequence characteristics is not exactly known, the term apparent tissue sodium concentration (aTSC) is more appropriate.^18^, ^19^

Thus, the aims of this study were to: 1) determine ^23^Na relaxation times for ex‐vivo human IVDs; and 2) investigate the correlation between MRI‐measured aTSCs in ex vivo IVDs and their histological classification assessed by Thompson grading.^20^ The study hypothesis was that increasing disc degeneration and increasing Thompson grade would result in decreasing aTSC levels.

## Materials and Methods

### Study Population

The human lumbar spine cadavers of 11 body donors were prepared by the local Institute of Anatomy I (Heinrich Heine University, Düsseldorf, Germany) for this study. The specimens were provided in frozen state and before MRI measurements, the specimens were thawed and warmed to room temperature for at least 24 hours. For MR imaging, the specimens were not fixated in formaldehyde. The mean age of body donors at their time of death was 86 ± 8 years (range 74–101 years) and four were female while seven were male. Written informed consent was obtained from all body donors for their bodies to be used for medical research in general after death. The study was approved by the local ethics committee (Ethics Committee of Heinrich‐Heine‐University of Düsseldorf, study number: 2021‐1528).

### MRI

All MRI imaging was conducted using a 3 T MRI scanner (Siemens MAGNETOM Prisma, Siemens Heathineers, Erlangen, Germany) in combination with a dual‐tuned ^23^Na/^1^H transmit receive surface coil (RAPID Biomedical GmbH, Rimpar, Germany). The coil consisted of an 11 cm circular single loop ^23^Na resonator and an 18 cm × 24 cm rectangular single loop ^1^H resonator. All images were acquired using a density‐adapted 3D radial sequence (DA‐3D‐RAD).^21^ This 3D imaging sequence was developed by Nagel et al and employs a density‐adapted radial sampling, which enables imaging of tissues with short echo times and improved SNR compared to conventional 3D radial sequences.^21^ The cadaver specimens were positioned on their side with the theoretical head toward the MRI scanner. To ensure that each IVD was measured with an acceptable coil sensitivity and thus also SNR, the dual‐tuned coil was placed on each IVD individually. Of the 11 cadaver specimens, a mean ± standard deviation of 3 ± 1 IVDs was measured each.

To determine aTSCs, three cylindrical reference phantoms (1.5 cm diameter, 10 cm length) were placed in close proximity to the examined IVD within the field of view. The phantoms were manufactured with different ^23^Na concentrations (50 mM, 100 mM, and 200 mM). To shorten the relaxation times of the phantoms, a fixed agarose content of 4% (ROTI®Garose, Carl ROTH GmbH & Co. KG, Karlsruhe, Germany) was used.^22^, ^23^, ^24^

### ^23^Na Coil Sensitivity Correction

The sensitivity of the surface coil is highly dependent on the distance of the coil from the examined tissue.^25^ The ^23^Na coil sensitivity was assessed by measuring a large cylindrical water phantom (18 cm diameter, 11 cm height) with a homogenous ^23^Na concentration of 154 mM. In the imaging protocol a repetition time (TR) of 15 msec and an echo time (TE) of 0.3 msec was used. Further imaging settings are documented in Table 1. To correct the coil sensitivity in each voxel, the signal was normalized. Each voxel of the following specimen measurements was divided by the corresponding normalized value of the sensitivity profile.

**TABLE 1:** MRI Sequence Parameters for Determining ^23^Na Coil Sensitivity and Measuring ^23^Na Relaxation Times (*T*_1_ and *T*_2_*) and Apparent Tissue Sodium Concentrations (aTSC) of the Specimen IVDs.

	Coil Sensitivity	^23^Na Relaxation Protocols		^23^Na Concentration	^1^H Imaging
	Protocol	^23^Na *T*_1_	^23^Na *T*_2_*	(aTSC) Protocol	Protocol
Sequence type	DA‐3D‐RAD	DA‐3D‐RAD	DA‐3D‐RAD	DA‐3D‐RAD	DA‐3D‐RAD
Nucleus	^23^Na	^23^Na	^23^Na	^23^Na	^1^H
Orientation	Sagittal	Sagittal	Sagittal	Sagittal	Sagittal
Repetition time (msec)	15	11/12/13/14/15/17/20/25/30/40/60	60	30	10
Echo time (msec)	0.3	0.3	[0.3/6.5/12.6/18.8] [1.5/7.7/13.8/20.0] [3.0/9.2/15.3/21.5]	0.1	1.0
Field of view (mm)	180 × 180 × 180	180 × 180 × 180	180 × 180 × 180	180 × 180 × 180	180 × 180 × 180
Projections	50,000	9000	9000	50,000	50,000
Voxel size (mm^3^)	2.0 × 2.0 × 2.0	3.0 × 3.0 × 3.0	3.0 × 3.0 × 3.0	2.0 × 2.0 × 2.0	0.5 × 0.5 × 0.5
Flip angle (°)	90	90	90	90	10
Pulse duration (msec)	0.50	0.50	0.50	0.15	0.50
Readout time (msec)	5	5	5	5	5
Averages	20	1	1	1	1
Total scan time (h:min:s)	04:10:00	00:38:33	00:27:00	00:25:00	00:08:20

DA‐3D‐RAD, density‐adapted 3D radial.

### Specimen Imaging Protocols

For the determination of the ^23^Na relaxation times of the IVDs, one IVD in each specimen was targeted. The IVD chosen was the healthiest IVD in the specimen, based on total height and overall appearance after initial ^1^H imaging, as this IVD should yield the highest SNR for ^23^Na‐imaging and subsequent ^23^Na relaxation time calculation.^11^ For ^23^Na *T*_1_ and *T*_2_* determination, 11 different TR and 12 different TE settings were used at a voxel size of 3 mm × 3 mm × 3 mm, which resulted in total scan times of 38:33 minutes:seconds and 27:00 minutes:seconds, respectively.

The aTSCs were measured with a separate imaging protocol. Each IVD of the 11 specimens was separately targeted by placing the middle of the coil on the IVD and the imaging protocol was repeated each time. A relatively small voxel size of 2 mm × 2 mm × 2 mm was chosen to reduce the influence of partial volume effects on the calculation of aTSC. To this end, a comparatively short TE of 0.1 msec and TR of 30 msec were used in order to improve the SNR with the acquisition of 50,000 projections in a total scan time of 25 minutes. To enable this short TE and reduce signal loss during excitation, an excitation pulse duration of 0.15 msec was used, as with the DA‐3D‐RAD the TE is defined from the middle of the excitation pulse to the start of the readout.

The ^1^H imaging protocol was also repeated for each IVD to determine coil positioning and to define regions‐of‐interest (ROIs) for ^23^Na parameter calculation. All images were reconstructed using a Hann Filter to reduce Gibbs ringing and to increase SNR. The settings of the protocols are listed in detail in Table 1.

### Image Post‐Processing

**FIGURE 1:**
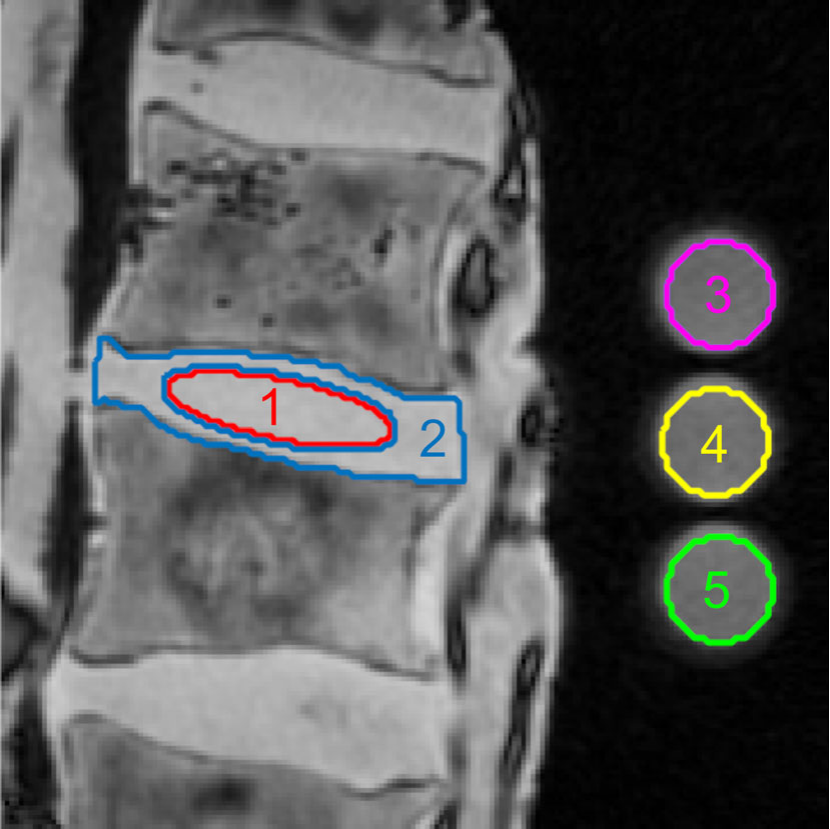
Example region‐of‐interest (ROI) placement illustrated by overlaying the ROI boundaries onto the respective ^1^H image. ROI number 1 corresponds to the nucleus pulposus (NP), ROI number 2 encapsulates the annulus fibrosus (AF), ROIs number 3, 4, and 5 are the reference phantoms with the ^23^Na concentrations 200 mM, 100 mM, and 50 mM. It is important to note, that ROI number 2 does not include the area of ROI number 1, because the NP is not part of the AF. In the sagittal slices, the innermost 60% of the intervertebral disc (IVD) was defined automatically using a matlab script as NP in elliptical shape, whereas the rest of the IVD was defined as AF.

The ROIs for the IVDs were defined on the higher resolution ^1^H MRI images with the software ITK‐SNAP (v3.8.0, Cognitica, Philadelphia, PA, USA).^26^ To this end, the outer contour of the whole IVD was outlined and aTSC was calculated for the central 25% of the IVD ROI. Limiting the assessment to the central part of the IVD was necessary for comparison with Thompson scoring which is only based on the central part of the IVD. Further processing of the image data was conducted using in‐house developed MATLAB (MathWorks, Natick, MA, USA, R2022a) scripts. Consistent with previous studies, in the sagittal slices, the innermost 60% of the IVD was defined automatically using a matlab script as NP in elliptical shape, whereas the rest of the IVD was defined as AF.^27^, ^28^ The ROIs were drawn independently once by B.K. (physicist, 6 years of experience in musculoskeletal imaging research) under supervision of D.B.A. (radiologist, 8 years of experience in musculoskeletal imaging) and once by M.F. (radiologist, 8 years of experience in musculoskeletal imaging). The second set of ROIs was used for assessing inter‐reader reliability, the rest of the results is exclusively presented for the first set of ROIs. The ROI placement is illustrated in Fig. 1.

The ^23^Na relaxation times were only calculated for the whole IVD, because the low resolution of the relaxation measurements would not allow reliable spatial distinction between NP and AF. *T*_1_ of ^23^Na was determined by fitting the TR variable signal values *S*(TR) of the relaxation protocol according to the following relation:

(1) $$S\left(\mathrm{TR}\right)=S_{0}∙\left(1-e^{\frac{-\mathrm{TR}}{T_{1}}}\right)+\text{noise}.$$

^23^Na *T*_2_* values were calculated by fitting the TE variable signal *S*(TE) biexponentially, because ^23^Na has a nuclear spin of 3/2 and therefore exhibits a short (*T*_2s_*) and a long (*T*_2l_*) transversal relaxation time component in biological tissue according to the following relation^29^:

(2) $$S\left(\mathrm{TE}\right)=S_{0}∙\left(p_{s}∙e^{\frac{-\mathrm{TE}}{T_{2s}^{*}}}+\left(1-p_{s}\right)∙e^{\frac{-\mathrm{TE}}{T_{2l}^{*}}}\right)+\text{noise}.$$

The parameter *p*_*s*_ indicates the proportion that the short relaxation time *T*_2s_* has in the total transversal relaxation and it satisfies the condition 0 < *p*_*s*_ < 1.

The aTSCs were determined for NP, AF, and the whole IVD. They were calculated by defining ROIs for the three agarose reference phantoms and fitting their concentration linearly against their mean ^23^Na signal to obtain a conversion factor for each voxel of the IVD. Furthermore, the aTSC was corrected for the difference in ^23^Na relaxation times between the agarose phantoms and the IVD tissue, which would otherwise influence the signal ratio between the phantoms and the IVDs. The following equation can be used to calculate the signal of the tissue *S*_*t*_(TR, TE) and phantoms *S*_*p*_(TR, TE) depending on their relaxation times and the TE and TR of the imaging sequence^17^:

(3) $$S_{t/p}\left(\mathrm{TR},\mathrm{TE}\right)=S_{0}∙\left(1-e^{\frac{-\mathrm{TR}}{T_{1}}}\right)∙\left(p_{s}∙e^{\frac{-\mathrm{TE}}{T_{2s}^{*}}}+\left(1-p_{s}\right)∙e^{\frac{-\mathrm{TE}}{T_{2l}^{*}}}\right).$$

By calculating the fraction $\frac{S_{t}\left(\mathrm{TR},\mathrm{TE}\right)}{S_{p}\left(\mathrm{TR},\mathrm{TE}\right)}$ a value is received, which is used to correct for different relaxation behavior of tissue and agarose reference phantoms. The agarose phantoms were manufactured according to a previously described procedure; hence, previously published relaxation parameters were assumed for the phantoms (*T*_1_ = 38.6 msec; *T*_2s_* = 4.5 msec; *T*_2l_* = 15.3 msec; *p*_*s*_ = 66.8%; *p*_*s*_ is defined according to Eq. 2).^30^ For the IVDs, the calculated ^23^Na relaxation values of the healthiest IVD of each specimen were used.

### Thompson Scoring

After MR imaging, the IVDs were analyzed by the local Institute of Anatomy I. The specimens were first decalcified and fixed in Ossa fixona (Diagonal, Münster, Germany), then dehydrated and embedded in paraffin. Afterwards the IVDs were cut along the mid‐sagittal plane and the condition of each IVD was semi‐quantitatively scored by T.J.F. (35 years of experience in musculoskeletal histopathology). The scoring was based on that of Thompson et al, assigning each IVD a grade between 1 and 5, with grade 1 representing no degeneration and grade 5 representing a fully degenerative IVD.^20^

As described by Thompson et al, the Thompson grade is assessed without staining of the tissue.^20^ In summary, Thompson grades are determined according to the following criteria^20^:

- Grade 1: Bulging gel in the NP and discrete fibrous lamellas in the AF
- Grade 2: White fibrous tissue peripherally in the NP and Mucinous material in the lamellas in the AF
- Grade 3: Consolidated fibrous tissue in the NP and extensive mucinous infiltration in the AF as well as loss of anular‐nuclear demarcation
- Grade 4: Horizontal clefts parallel to the endplate in the NP and focal disruptions in the AF
- Grade 5: Clefts extending though NP and AF

### Statistical Analysis

For all statistical analysis the software SPSS (IBM Corp. Released 2020. IBM SPSS Statistics for Windows, Version 27.0. Armonk, NY, USA: IBM Corp.) was used. Mean and standard deviation of the ^23^Na relaxation times of all measured IVDs were calculated. Mean and standard deviation aTSCs of the IVDs were also calculated according to their Thompson grade and are depicted in the following as “mean ± standard deviation.” The two sets of ROIs were used to assess inter‐reader reliability for aTSC measurements by calculating average intraclass correlation coefficients (aICC). The aICCs were categorized according to Koo et al.^31^ The values for aTSC of AF and NP were tested for significant difference in their respective Thompson grade using a Wilcoxon test with significance level *P* < 0.05. Furthermore, a Kendall Tau correlation was conducted and the rank correlation coefficient *τ* was calculated to investigate the relation between Thompson grade and aTSC. The significance level was set to *P* < 0.05 and the correlation was assessed as “low” for 0.1 ≤ |*τ*| < 0.3, “moderate” for 0.3 ≤ |*τ*| < 0.5, and “high” for |*τ*| ≥ 0.5.^32^

## Results

**FIGURE 2:**
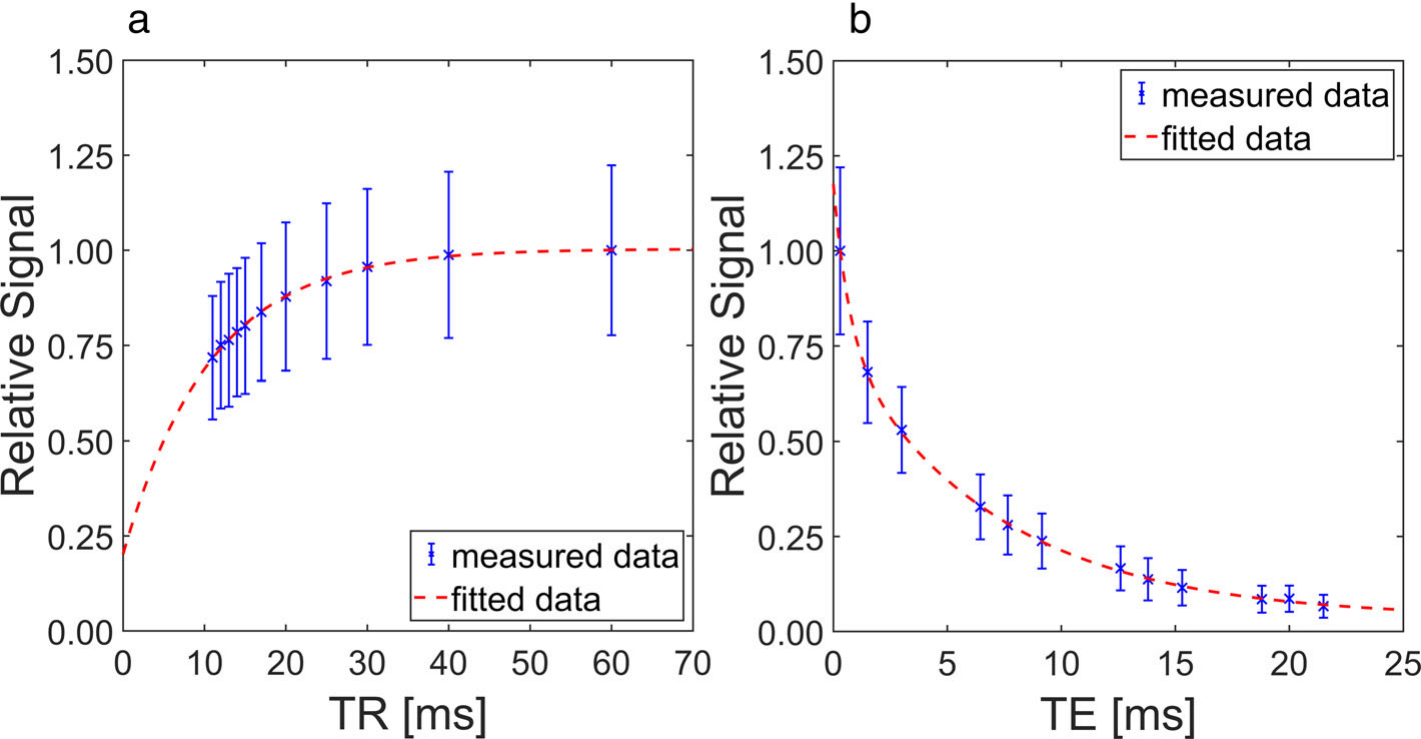
Example fits of the ^23^Na relaxation time data of an intervertebral disc (IVD) with a Thompson grade of 2. (**a**) For the determination of *T*_1_, a monoexponential fit was performed, resulting in ^23^Na *T*_1_ = 10.7 msec and *R* ^2^ = 0.998. (**b**) The data for *T*_2_* was fitted biexponentially due to the properties of the ^23^Na nucleus, resulting in the values ^23^Na *T*_2s_* = 0.7 msec, ^23^Na *T*_2l_* = 7.0 msec, *p*_*s*_ = 35.2%, and *R* ^2^ = 0.999. The parameter *p*_*s*_ indicates the proportion that the short relaxation time *T*_2s_* has in the total transversal relaxation.

^23^Na relaxation times were able to be determined for all imaged IVDs. The mean values for *T*_1_‐fitting were ^23^Na *T*_1_ = 9.8 ± 1.3 msec with coefficient of determination (*R* ^2^) = 0.981 ± 0.018 and for *T*_2_*‐fitting, the mean values were ^23^Na *T*_2s_* = 0.7 ± 0.1 msec, ^23^Na *T*_2l_* = 7.3 ± 1.1 msec and *p*_*s*_ = 32.7 ± 4.0% with *R* ^2^ = 0.999 ± 0.001. In Fig. 2, the fits of the relaxation protocol data of one example IVD are shown.

Of the 30 IVDs, 3, 4, 5, 5, and 13 had Thompson grades of 1, 2, 3, 4, and 5, respectively. This indicates a clear trend toward increased Thompson and thus degradation grades for the IVDs from our study. The aTSC values of the IVDs decreased with increasing Thompson grade, the only exception being the transition from Thompson grade 3 to 4 for NP, AF and the whole IVD. In general, the aTSC values for NPs were higher than the values for AFs in their respective Thompson grades. This difference was not significant for Thompson grades 1 (*P* = 0.109) and 2 (*P* = 0.068), but it was significant for Thompson grades 3–5.

**FIGURE 3:**
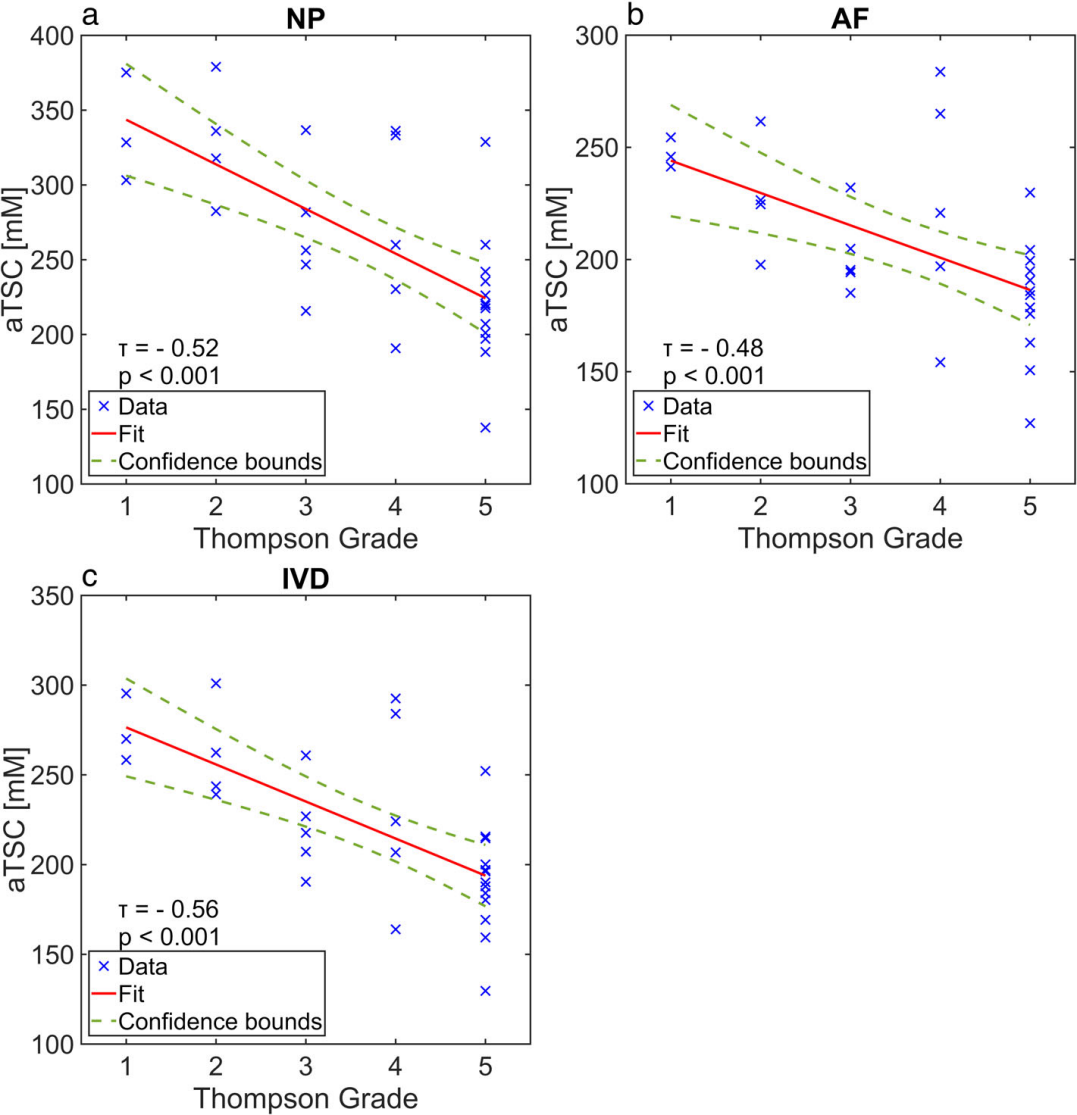
Scatter plots with linear regressions of calculated aTSCs and their respective Thompson grades for (**a**) NP, (**b**) AF, and (**c**) the whole intervertebral disc (IVD). The Kendall Tau test resulted in a strongly negative correlation for (a) the NP (*τ* = −0.52) and (c) the whole IVD (*τ* = −0.56). For (b) the AF the Kendall Tau test resulted in a moderately negative correlation (*τ* = −0.48).

For the NP, the mean aTSC values decreased from 335.6 ± 36.5 mM for Thompson grade 1 to 221.6 ± 43.8 mM for Thompson grade 5. The mean aTSC values for the AF decreased from 247.2 ± 6.6 mM for Thompson grade 1 to 182.3 ± 24.5 mM for Thompson grade 5. The mean aTSC values for the whole IVD decreased from 274.6 ± 18.9 mM for Thompson grade 1 to 190.5 ± 29.5 mM for Thompson grade 5. The inter‐reader reliability was calculated to be aICC = 0.995 for the NP, aICC = 0.976 for the AF, and aICC = 0.988 for the whole IVD, indicating excellent reliability. The results are listed in detail in Table 2. Scatter plots with linear regressions of the aTSC values and their corresponding Thompson grades are shown in Fig. 3.

**TABLE 2:** Mean Values and Standard Deviations of aTSCs for the Nucleus Pulposus (NP), Annulus Fibrosus (AF) and Whole IVDs According to Their Assigned Thompson Grades. Furthermore, the *τ*‐values for the Kendall‐Tau‐Correlation tests are listed and the bold values indicate a significant correlation. Thereafter, the average intraclass correlation coefficient (aICCs), with which inter‐reader reliability was assessed, are listed.

Thompson Grade	Number of IVDs	aTSC (NP) (mM)	aTSC (AF) (mM)	aTSC (IVD) (mM)
1	3	335.6 ± 36.5	247.2 ± 6.6	274.6 ± 18.9
2	4	328.8 ± 40.2	227.6 ± 26.2	261.5 ± 28.2
3	5	267.4 ± 45.3	202.3 ± 18.0	220.6 ± 26.2
4	5	270.1 ± 63.9	224.1 ± 52.1	234.3 ± 54.0
5	13	221.6 ± 43.8	182.3 ± 24.5	190.5 ± 29.5

	NP	AF	IVD
*τ*‐value Kendall‐Tau‐Correlation	**−0.52**	**−0.48**	**−0.56**
aICC inter‐reader reliability	0.995	0.976	0.988

**FIGURE 4:**
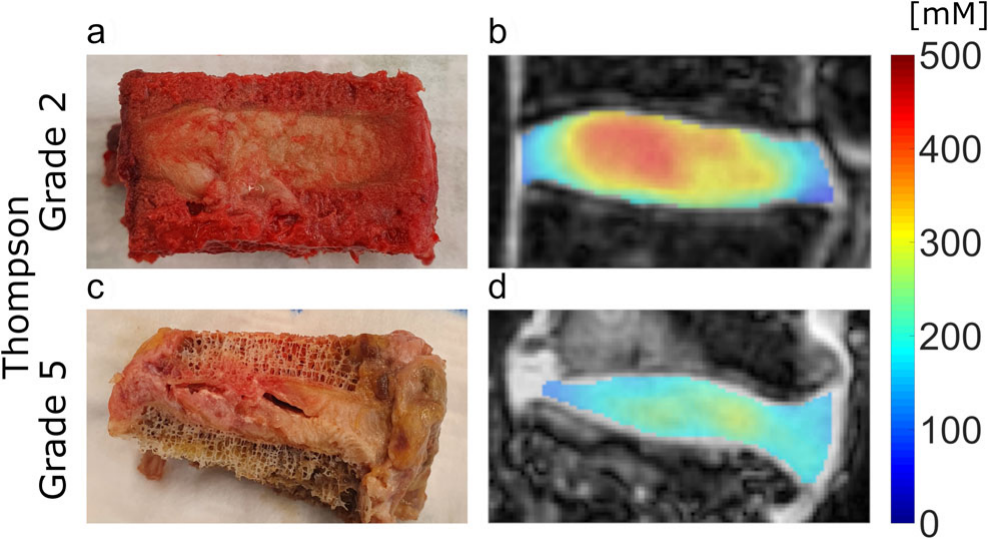
(**a**, **c**) Anatomical images for Thompson grading and (**b**, **d**) aTSC overlays onto the acquired ^1^H MRI images. Figure (a) shows an intervertebral disc (IVD) with a Thompson grade of 2 and a mean aTSC of 371.7 ± 42.4 mM for the NP, 258.7 ± 37.4 mM for the AF, and 298.0 ± 92.7 mM for the whole IVD. Figure (c) shows an IVD with a Thompson grade of 5 and a mean aTSC of 201.1 ± 45.8 mM for the NP, 163.0 ± 46.6 mM for the AF, and 169.2 ± 48.5 mM for the whole IVD. Figures (b) and (d) present the corresponding MR images.

The Kendall Tau correlation test resulted in a significant and highly negative (*τ* = −0.52) correlation for the aTSC values and Thompson grades of the NP. The correlation for the values of AF was also significant and was moderately negative (*τ* = −0.48), whereas the correlation for the values of the whole IVD was significant and highly negative (*τ* = −0.56). In summary, the *τ*‐values of these correlations had very little difference between each other. In Fig. 4, example images for Thompson grading and overlays of aTSC maps on ^1^H images are shown.

## Discussion

In this study, ^23^Na relaxation times and aTSCs were measured in human ex vivo IVDs. For ^23^Na relaxation times in IVDs, data are scarce. Wang et al reported ^23^Na *T*_1_ and monoexponential *T*_2_* relaxation times of 22 msec and 16 msec, respectively, in bovine ex vivo IVDs.^6^ The study used progressive saturation acquisitions for the determination of *T*_1_ and varying TE acquisitions for the calculation of *T*_2_*. Their measured *T*_1_ is approximately twice as high as that for human IVDs in the current study. The *T*_2_* is also approximately a factor of two higher than the *T*_2l_* calculated for human IVDs in the current study. Detailed measurement parameters were not provided by Wang et al and, in particular, no mention was made of why ^23^Na *T*_2_* was determined via a monoexponential fitting process.

One plausible reason for the monoexponential fitting of the ^23^Na *T*_2_* time could be that Wang et al used a vendor provided 3D fast low angle shot (FLASH) sequence with a TE of 6 msec.^6^ This would suggest that they may not have had the data required to measure the *T*_2s_* of IVDs, as the *T*_2s_* signal would have largely decayed in measurements with TE >1 msec. Thus, their measured parameter *T*_2_* = 16 msec should be close to the *T*_2l_* = 7.3 ± 1.1 msec value measured in the current study, which it is not.

The difference between their measured *T*_2_* and the *T*_2l_* in the current study could be due to differences between bovine and human IVDs. A previous study has reported that bovine and human IVDs may not have the same tissue properties.^33^ Nonidentical tissue properties may also explain the difference between the longitudinal ^23^Na relaxation times between the bovine and human studies. Furthermore, different temperatures of the IVDs could lead to different relaxation times.^34^ Wang et al did not specify the temperature at which the bovine IVDs were imaged, whereas the human IVDs in the current study were imaged at room temperature.

In the study of Çavuşoğlu et al, the in vivo TSCs of human IVDs of five healthy volunteers were between 254.6 ± 54 mM and 290.1 ± 39 mM, depending on the position of the IVD.^35^ These values are in good agreement with the aTSC values of the less damaged (Thompson grade 1–2) IVDs in the current study. The aTSCs of the IVDs with Thompson grade 3–5 were lower than the values of Çavuşoğlu and his coworkers. This could indicate that aTSCs decrease with increasing degradation of the IVDs, but Çavuşoğlu et al only measured healthy volunteers.

Malzacher et al measured a TSC of 327 ± 53 mM for IVDs in the lumbar region, which is slightly higher than the aTSC of 274.6 ± 18.9 mM we calculated for the healthiest IVDs with Thompson grade 1.^13^ Moon et al examined the TSC for the lumbar IVDs in a rabbit model and calculated a TSC of 269.7 ± 6.3 mM for the whole IVD.^12^ These values also align well with our aTSCs for the relatively healthy IVDs of Thompson grade 1–2.

The lower aTSC values in the AF compared to the NP in the current study are likely caused by the differing GAG concentrations between the two regions.^36^ Although this difference was not significant for the IVDs with Thompson grade 1 and 2, this is likely caused by the low number of data points (3 and 4 points respectively) in the test.

PG, with its GAG side chains, accounts for approximately 50% of the dry weight in the NP and 10%–20% in the AF.^36^ Differences between NP and AF based on varying GAG concentrations have been shown using biochemically sensitive MRI imaging like CEST.^27^ However, in the current study, the measured aTSC did not increase linearly with the expected GAG content between AF and NP. Wang et al compared TSC directly with PG content in bovine IVDs and showed that aTSC increased linearly with PG content.^6^ In the current study, the mean aTSC of the NP with Thompson grade 1 was approximately 50% higher than that of the AF in this grade, while the PG content in the healthy IVD is reported to be 2.5 times higher.^36^ One reason could be partial volume effects, to which ^23^Na imaging is prone due to its inherently low imaging resolution.^37^ Another reason could be age related changes in PG content, as the body donors of the current study were relatively old at their time of death (86 ± 8 years) and age has been shown to influence the PG content of NP and AF.^38^

In their ex vivo study, Wang et al correlated the ^23^Na concentration in bovine IVDs with the corresponding histologically determined PG content and found a significant positive correlation (*r* = 0.71).^6^ This suggested the suitability of aTSC determination for estimating the health of the IVDs, as a decrease in PG and GAG, which as a side chain of PG is strongly associated with it, has been reported with increasing degrees of degradation of human IVDs.^39^ The current study showed a negative correlation between aTSCs and Thompson grading. This relationship was expected and supports the MRI assessment of aTSCs for investigating IVD status.

Our methodology would have to be altered slightly to assess aTSC of IVDs in vivo. The imaging protocol as a whole would have to be shortened and, likely, only one IVD would be possible to be examined per imaging session, as it would not be reasonable to have a scanner time of over 1 hour for volunteers and patients. Other approaches to accelerate imaging could include advanced image reconstruction techniques like compressed sensing or MR fingerprinting for aTSC calculation and relaxation time measurements.^17^ Another challenge could be the sensitivity profile of the surface coil, as the sensitivity declines quickly with increasing distance to the coil. In vivo, the distance of the IVDs would be greater than in our ex vivo measurements, limiting the sensitivity of the coil at the position of the IVD and necessitating even more careful correction of coil sensitivity effects.^25^

## Limitations

First, the IVD ^23^Na relaxation times were assessed at room temperature. ^23^Na relaxation times have been shown to change with the temperature of the examined tissue,^34^ hence in future ex vivo studies, the specimens should be warmed to the human body temperature of 37°C. However, this may accelerate decay of the ex vivo specimens during the long imaging protocols.

Second, the tissue properties and consequently the measured ^23^Na relaxation times of the IVD specimens could be additionally influenced by the freeze and thaw cycles, which were necessary for the preservation of the ex vivo samples. This alteration of biomechanical properties through freeze and thaw cycles has been shown for porcine IVD tissue and could also influence the human IVD specimens used in this study.^40^

Third, in the estimation of aTSC values, the ^23^Na relaxation times of IVDs and agarose phantoms were used to correct for different ^23^Na signal intensities due to relaxation effects. ^23^Na relaxation times have been shown to change with increasing degradation in the articular cartilage of the patella in vivo in humans.^23^ In the current study, only the healthiest of the IVDs for each specimen was imaged for ^23^Na relaxation time assessments because the imaging protocols per sample were already long. However, if the ^23^Na relaxation times change with increasing degradation, this could make the relaxation correction less accurate for the IVDs with higher Thompson grade. In this study, the IVDs with a high Thompson grade (Thompson grade 4–5: N = 18) were more numerous than the IVDs with low Thompson grade (Thompson grade 1–2: N = 7).

Fourth, for the correlation between aTSC and Thompson grade, a uniform distribution of Thompson grades as well as a higher number of IVDs in general for more expressive statistics would be desirable. In our case, this would mean including more IVDs with lower Thompson and degradation grade and, thus, more IVDs with morphologically healthy properties. The relatively high number of degraded IVDs in the current study was likely caused by the high age of our body donors at their time of death (86 ± 8 years). Furthermore, a higher number of IVDs in each Thompson degradation grade would enable further statistical testing, like testing for significant difference between the aTSCs of each Thompson degradation grade.

Fifth, the degradation grade was not compared to established MRI grading systems like the Pfirrmann score.^11^ However, for the image quality of the *T*_2_‐weighted images to be sufficient for Pfirrmann scoring, the imaging coil would have to be changed to a conventional 1H multi‐array acquisition coil. This would have elongated the already lengthy imaging sessions even further, which could have led to more decomposition of the cadaver specimens before Thompson grading.

## Conclusion

In this study, the ^23^Na relaxation times were measured in ex‐vivo human IVDs and used to determine aTSCs. The results showed a strong negative correlation between the measured aTSC and the degeneration of the IVD, which was assessed by histological Thompson grading.
